# Kidney-Specific Reduction of Oxidative Phosphorylation Genes Derived from Spontaneously Hypertensive Rat

**DOI:** 10.1371/journal.pone.0136441

**Published:** 2015-08-26

**Authors:** Jason A. Collett, Jiffin K. Paulose, Vincent M. Cassone, Jeffrey L. Osborn

**Affiliations:** Department of Biology, University of Kentucky, Lexington, KY, the United States of America; University Medical Center Utrecht, NETHERLANDS

## Abstract

Mitochondrial (Mt) dysfunction contributes to the pathophysiology of renal function and promotes cardiovascular disease such as hypertension. We hypothesize that renal Mt-genes derived from female spontaneously hypertensive rats (SHR) that exhibit hypertension have reduced expression specific to kidney cortex. After breeding a female Okamoto-Aoki SHR (SAP = 188mmHg) with Brown Norway (BN) males (SAP = 100 and 104 mmHg), hypertensive female progeny were backcrossed with founder BN for 5 consecutive generations in order to maintain the SHR mitochondrial genome in offspring that contain over increasing BN nuclear genome. Mt-protein coding genes (13 total) and nuclear transcription factors mediating Mt-gene transcription were evaluated in kidney, heart and liver of normotensive (NT: n = 20) vs. hypertensive (HT: n = 20) BN/SHR-mt^SHR^ using quantitative real-time PCR. Kidney cortex, but not liver or heart Mt-gene expression was decreased ~2–5 fold in 12 of 13 protein encoding genes of HT BN/SHR-mt^SHR^. Kidney cortex but not liver mRNA expression of the nuclear transcription factors *Tfam*, *NRF1*, *NRF2* and *Pgc1α* were also decreased in HT BN/SHR-mt^SHR^. Kidney cortical tissue of HT BN/SHR-mt^SHR^ exhibited lower cytochrome oxidase histochemical staining, indicating a reduction in renal oxidative phosphorylation but not in liver or heart. These results support the hypothesis that renal cortex of rats with SHR mitochondrial genome has specifically altered renal expression of genes encoding mitochondrial proteins. This kidney-specific coordinated reduction of mitochondrial and nuclear oxidative metabolism genes may be associated with heritable hypertension in SHR.

## Introduction

Hypertension constitutes a primary and significant factor in the development of cardiovascular disease. Despite major gains in the long-term treatment of hypertension, cardiovascular disease remains the number one cause of death and disability in developed countries. Primary or essential hypertension is regarded as a multi-factorial disease, influenced by both genetic inheritance and environmental conditions that influence gene expression. The genetic basis of hypertension has been focused primarily on inheritance and expression of nuclear genes (nDNA) [1], despite the fact that mitochondria are present in multiple copies in each cell and have their own genome. Oxidative phosphorylation (OXPHOS) depends on the coordinated expression of two separate but interactive genomes, nuclear and mitochondrial. Numerous trans-factors involved with mtDNA replication, transcription and mRNA processing are nuclear encoded, including mtRNA polymerase, mtDNA polymerase, several regulatory transcription factors and mtRNA processing proteins [2]. This nuclear-mitochondrial interaction is essential to cellular health and function, and therefore may play a large role in the development of or response to cardiovascular disease [3].

Mitochondrial dysfunction has been implicated in a wide variety of genetic disorders [4, 5], and alterations in mitochondrial function have been observed in conjunction with aging and development of hypertension in both rodents and humans [6–9]. Recently, mitochondrial transfer RNA (tRNA) mutations were observed in a genetically focused population with a high incidence of essential hypertension [10, 11]. Wilson et al. [12] have described a correlation between a T4921C transition SNP, which lies in the mitochondrial tRNA^lle^ gene (GenBank accession no. NC_001807) and hypertension. The genetic association of mtDNA variants and tRNA mutations [4] to type 2 diabetes and hypertension directly suggested mitochondrial dysfunction in the development of cardiovascular disease and metabolic syndrome [9]. Taken together, there is growing evidence that altered mitochondrial gene expression may have a significant role in the generation of cardiovascular disease phenotypes. The mechanisms by which mitochondrial genes are expressed and regulated in disease remains unclear.

The impact of the kidney on long-term blood pressure regulation is well known. It has been hypothesized that the “set-point” for the long-term control of BP resides in the kidney [13–15]. In this model, the set-point of the chronic renal function curve establishes the steady state relationship between renal perfusion pressure and urinary excretion of sodium and water, which in turn affects blood volume and cardiac output. This renal-body fluid-pressure control system exhibits “infinite feedback gain”, i.e., BP will stabilize only when intake and output of sodium and water become exactly equal, which occurs at one pressure level for any given renal function curve and salt intake level [16]. The importance of kidney function to modulate blood pressure has been shown by Lifton et al. [17] demonstrating genetic variants in important renal pathways underlie all of the Mendelian disorders affecting blood pressure homeostasis. These studies postulated that altered renal expression of genes may contribute significantly to the disease. The goal of the present study was to determine the renal expression of mitochondrial protein-encoding genes and the nuclear genes known to regulate their expression, in a unique rat strain with localized mt-DNA from Okomoto-Aoki SHR. The “conplastic” strain was developed by crossing a hypertensive female Spontaneously Hypertensive Rat (SHR) with normotensive, male Brown Norway (BN) rats (BN/SHR-mt^SHR^) [18]. Hypertensive female offspring were then phenotypically selected and crossed with founder males for several generations. All offspring had identical mitochondrial DNA (mtDNA) of the progenitor SHR, barring any mutation. The results of these studies document significant reduced expression of renal mtRNA and nuclear encoded regulatory elements in hypertensive male and female offspring. These studies suggest that specifically reduced renal cortical mtRNA expression may lead to decreased OXPHOS in rats with isolated mitochondrial SHR genomes and hypertension.

## Materials and Methods

### Experimental Animals

All experiments were carried out in strict accordance with the recommendations provided in the AAALAC Guide to the Care and Use of Laboratory Animals and the National Institutes of Health. All protocols in this study were specifically approved by the University of Kentucky Institutional Animal Care and Use Committee (IACUC #000649L2003). All surgeries were performed following complete anesthesia of individual animals. Tissue collection for these studies also was conducted under full anesthesia. At the time of any change in protocol (minor or major) amendments were submitted to the IACUC and approved prior to conducting any individual experiment. Since this was a long term breeding study, the original IACUC approval served for a significant time period. All individual studies for this protocol approval were reevaluated at 3 year intervals as required by AAALAC standards. A “conplastic” colony using phenotypic selection was employed. The development and phenotypic characterization have been described in detail elsewhere [18]. The Aoki-Okamoto SHR/Brown Norway (BN/SHR) rat colony was developed by breeding a female SHR (Charles River Labs, Wilmington, MA) with 2 different normotensive Brown Norway males (BN* and BN^, respectively; Charles River Labs, Wilmington, MA). Beginning at 10 weeks of age, rats were phenotyped using tail cuff plethysmography (Kent Scientific, Torrington, CT). Each individual animal was trained and acclimated to the tail cuff device prior to measuring blood pressure. In these training sessions all animals were covered and remained in the dark to provide the type of comfort that behaviorally meets the needs of rats. Following blood pressure determinations, the most hypertensive female offspring were then back-crossed to the original progenitor BN males for 5 subsequent generations. Animals were monitored daily for health and evaluated for normal behavioral activity.

After repeated blood pressure recordings that assured consistent determination of arterial pressure, rats not scheduled for rebreeding were euthanized with an overdose of sodium pentobarbital (60 mg/kg i.p), decapitated, and organ tissues were rapidly frozen in acetone super-cooled on dry ice and stored for later analysis.

### Measurement of arterial pressure

**Fig 1:**
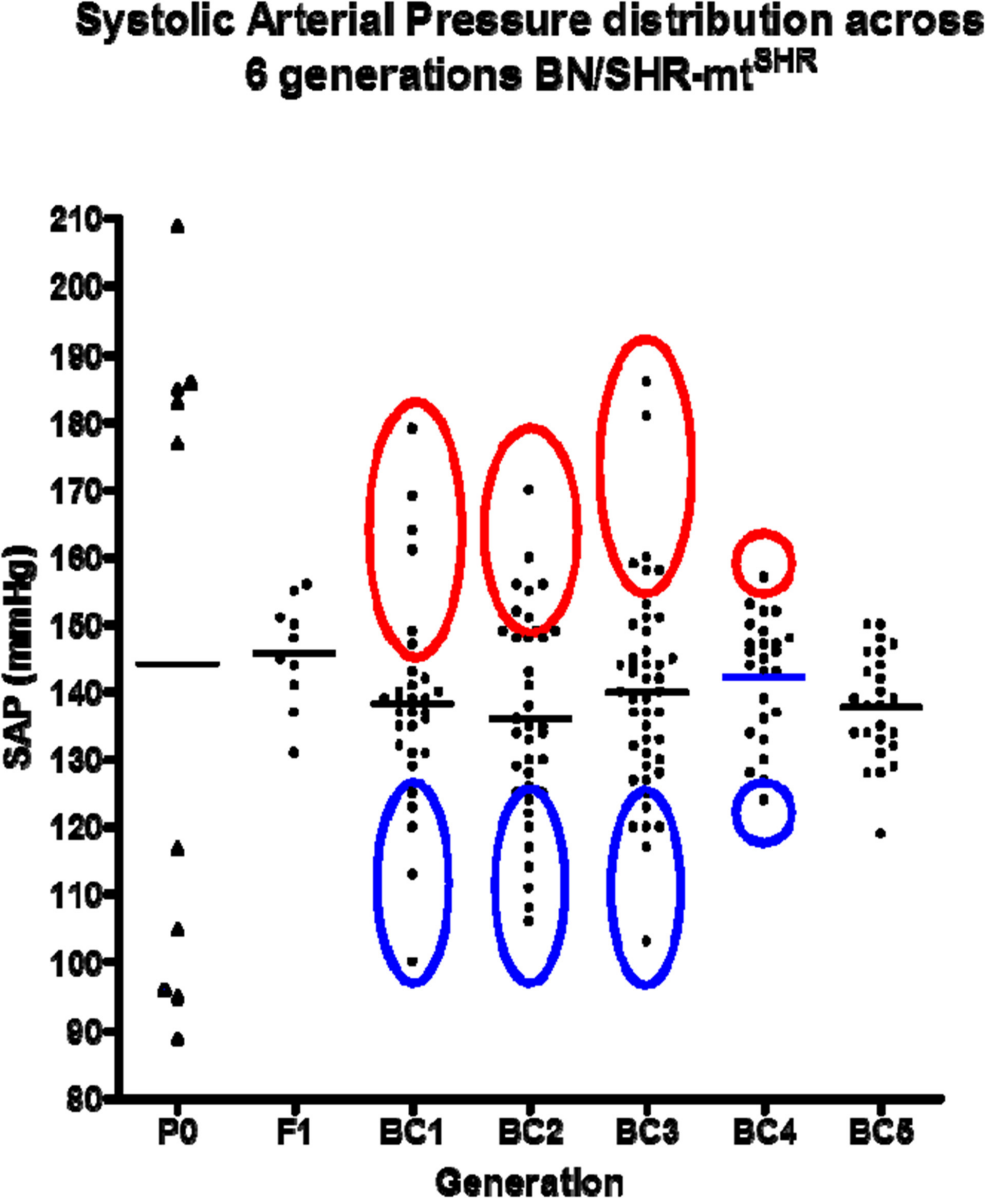
Six generations of BN/SHR-mt^SHR^ rats with corresponding average systolic pressure (SAP) values. Three distinct populations persisted throughout all 6 generations, with hypertension being dominantly expressed and maintained despite the reduction of the SHR nuclear genome. Tissues from HT animals used in all genome analysis experiments (both mitochondrial and nuclear) are indicated by the red circles. Tissues used from NT animals are indicated by the blue circles (adapted from Collett et. al.; 10).

Systolic arterial pressure (SAP) was evaluated in parents and offspring beginning no earlier than 10–12 weeks of age. Phenotypes were assigned as either normotensive (NT: SAP ≤ 124mmHg), borderline hypertensive (BHT: 125 ≤ SAP ≤149 mmHg) or hypertensive (HT: SAP ≥ 150mmHg; Fig 1). To minimize stress and improve reliability of BP measurements, several steps were used in the BP recording method that has been previously characterized and published [19, 20]. Rats were exposed and acclimated to the measurement procedures and restraint equipment prior to BP recordings. A dark cover was placed over the restrained animal for the duration of the BP measurement, and BP recordings were performed at the same time each day. All equipments were thoroughly cleaned and disinfected before and after each individual rat to eliminate foreign scent. Animals were moderately warmed to dilate the ventral artery. Arterial pressures were derived from the average results of ≥5 measurements in each recording session. The average BP of ≥5 separate recording sessions with <5% variability was used to establish the phenotype of each animal. Both systolic and diastolic pressures were obtained and recorded. For purposes of reporting, the systolic pressures were used for the determination of the specific individual phenotype. NT (blue) and HT (red) age and sex-matched BN/SHR-mt^SHR^ rats (11 female/10 male per group) were utilized for the current study (Fig 1). There was no significant difference in BP between male and female NT rats or male and female HT rats (p>0.05).

### RNA Extraction and RT-PCR

Renal cortex, liver and left ventricular cardiac tissue were selected from age and sex-matched HT and NT BN/SHR-mt^SHR^ rats (n = 20 NT; n = 20 HT) as described above. Total RNA was extracted with Trizol reagent (Invitrogen, Carlsbad, CA) and purified using RNeasy minicolumns (Qiagen Inc., Valencia, CA) according to the manufacturer’s protocol. Possible genomic DNA was digested with DNase I (Qiagen Inc., Valencia, CA). Concentration and purity of all RNA samples was determined by the Nanodrop ND-1000 spectrophotometer (Nanodrop Technologies, Wilmington, DE). Extracted RNA was reverse-transcribed into complementary DNA (cDNA) using qScript cDNA Supermix (Quanta Biosciences, Gaithersburg, MD) in a total volume of 20μl using a MyCyler Thermal Cycler (Bio-Rad Laboratories, Hercules, CA).

### Quantitative Real-Time PCR

Quantitative Real-Time PCR was performed on a StepOnePlus Real-time PCR system (Applied Biosystems, Foster City, CA). Real-time quantitative PCR amplifications were performed in triplicate on a 96-well plate. Pre-designed TaqMan primers and hydrolysis probes for all genes of interest were purchased from Applied Biosystems (*mt-ND1*- Rn03296764_s1, *mt-ND2*- Rn03296765_s1, *mt-ND3*- Rn03296825_s1, *mt-ND4*- Rn03296781_s1, *mt-ND4L*- Rn03296792_s1, *mt-ND5*- Rn03296799_s1, *mt-ND6*- Rn03296815_s1, *mt-CO1*- Rn03296721_s1, *mt-CO2*- Rn03296737_s1, *mt-CO3*- Rn03296820_s1, *mt-CYB*- Rn03296746_s1, *mt-ATP6*- Rn03296710_s1, *mt-ATP8*- Rn03296716_s1, *NRF1*- Rn01455958_m1, *NRF2a*- Rn01767215_m1, *NRF2b*-Rn01514289_g1, *Pgc1α*-Rn00598552_m1, *Tfam*-Rn00580051_m1, *Cyc1*-Rn01504159_g1, *Cox6c*-Rn00820983_gH, *GAPDH*- Rn01775763_g1). Primers and probes were verified as operating at similar efficiencies. Target gene and endogenous control amplicons were labeled with either FAM or VIC. The levels of glyceraldehyde-3-phosphate dehydrogenase (*GAPDH*) expression were measured in all samples to normalize gene expression for sample-to-sample differences in RNA input, RNA quality and reverse transcription efficiency. Each sample was analyzed in triplicate, and the expression was calculated according to the 2^−ΔΔCt^ method [21, 22].

### Citrate Synthase Assay

Citrate synthase activity was determined in homogenates prepared from kidney tissue using a citrate synthase assay kit (CS0720; Sigma-Aldrich, St. Louis, MO) [23]. Total muscle protein was determined in triplicate by the method of Bradford [24] and the protein concentration of all samples was equalized. Citrate synthase activity was determined based on the formation of 2-nitro-5-thiobenzoic acid at a wavelength of 412 nm at 25°C on a microplate absorbance reader (iMark; BIO RAD, Hercules, CA). In each well, 8 μl of sample was added to a reaction medium containing 178 μl of assay buffer, 2 μl of 30 mmol/L acetyl coenzyme A, and 10 mmol/L 2-nitro-5-thiobenzoic acid. The baseline solution absorbance was recorded, reactions were initiated by the addition of 10 μl of oxaloacetic acid, and the change in absorbance measured every 15 seconds for 2 minutes.

### Cytochrome Oxidase Histochemistry

Cytochrome oxidase (CO) activity was determined in fresh frozen sections (20μm) in kidney, liver and heart tissue, as described previously [25]. Briefly, fresh frozen tissue was sectioned on a cryostat at 20 μm. Slides were immersed in 0.5% glutaraldehyde in 0.1% phosphate buffer for 5 minutes. Slides were then incubated for 2 hours in the same diaminobenzidene (DAB)/cytochrome c solution simultaneously (preceded by 5 minutes of sparged oxygen) at 37°C. Slides were then postfixed in 10% formalin for 15 minutes. Finally, slides were immersed in a serious of ethanol dehydration steps: 50, 70, 90, 95, 100% ethanol (30 seconds each) and xylene (2 changes, 5 minutes each). Slides were then coverslipped using Histomount (Life Technologies) and dried overnight. Colorometric change was used as a direct measurement of metabolic activity, in which darker color indicated higher metabolically active tissue. Relative density per area was calculated using ImageJ (NIH).

### Data analysis

Blood pressures and citrate synthase activity among animals were initially analyzed by 1-way analysis of variance (ANOVA) followed by post-hoc comparisons using the Bonferroni t-test. Tissue mRNA expression levels were analyzed using Mann-Whitney *U* Test comparisons. The 0.05 level of probability was utilized as the minimum criterion of significance. All statistical analyses were performed using GraphPad Prism 4.0 (GraphPad Software, Inc., La Jolla, CA).

## Results and Discussion

### Evaluation of mt-gene expression

Multiple mtDNA encoded genes of the mitochondrial respiratory chain were significantly reduced in renal, but not liver or cardiac tissue of HT BN/SHR-mt^SHR^, including five complex I, one complex III, three complex IV and both subunits of ATP synthase. Additionally, the nuclear trans-factors *Tfam*, *NRF1*, *NRF2a*, *NRF2b* and *Pgc1a* were all downregulated in the kidney, but not elsewhere, of HT BN/SHR-mt^SHR^.

#### Complex I: NADH dehydrogenase

**Fig 2:**
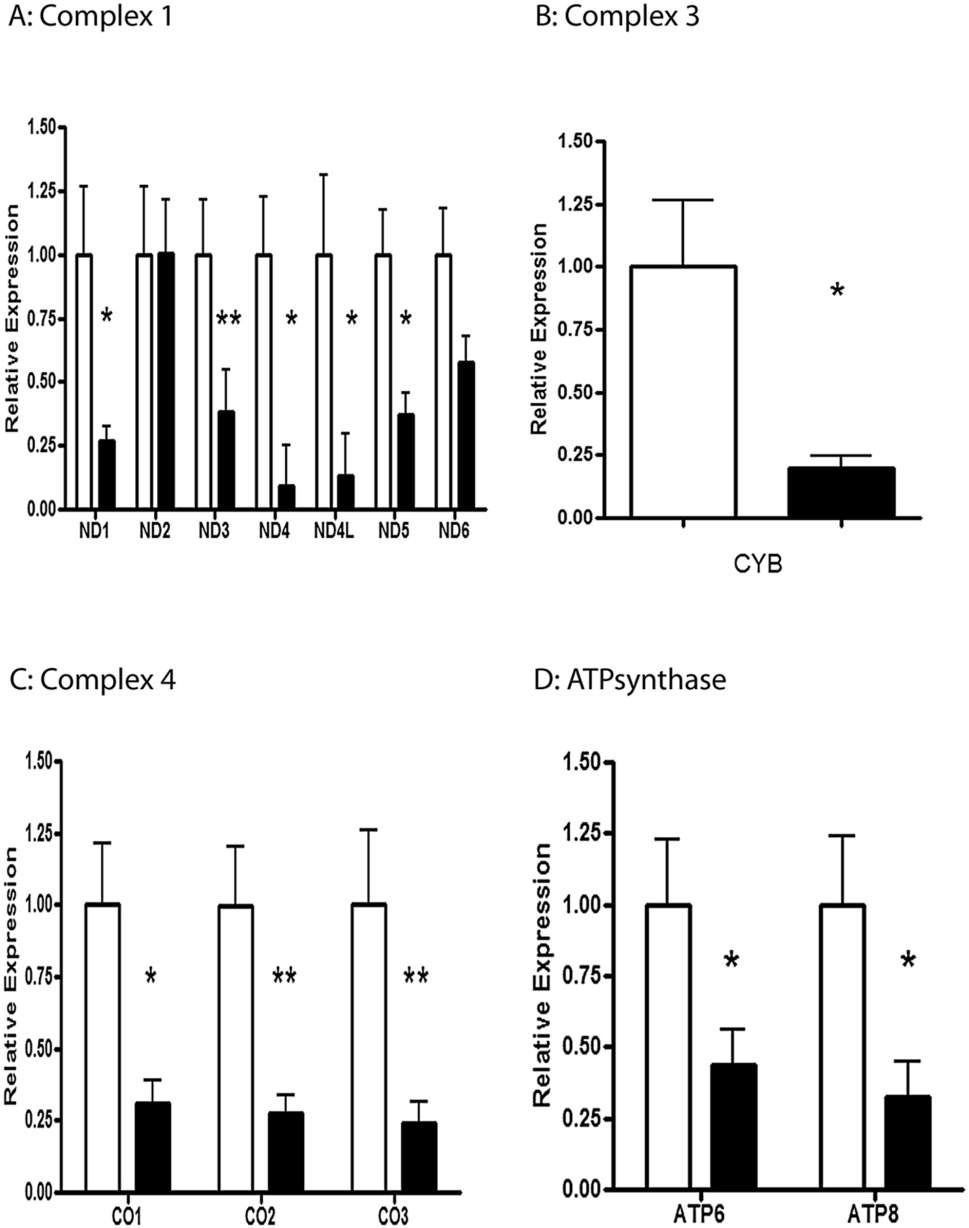
Quantitative Real-Time PCR mt-genes across 4 complexes of ETC. **A:** *mt-ND1* was reduced ~3.7 fold in HT BN/SHR-mt^SHR^ (*P<0.05). mt-*ND3* was reduced ~2.6 fold in HT SHR/BN-mt^SHR^ (**P<0.01). *mt-ND4* was reduced ~10.8 fold in HT SHR/BN-mt^SHR^ (*P<0.05). *mt-ND4L* was reduced ~7.7 fold in HT SHR/BN-mt^SHR^ (P<0.05). *mt-ND5* was reduced ~2.7 fold in HT SHR/BN-mt^SHR^ (P<0.05). *mt-ND6* was reduced ~1.7 fold in HT SHR/BN-mt^SHR^ (P>0.05) (Fig 1A). *mt-ND2* was not different between the two phenotypes (P>0.05). **B:** *mt-CYB* was reduced ~5 fold in HT BN/SHR-mt^SHR^ (*P<0.05). **C:** *mt-CO1* was reduced ~3.2 fold in HT BN/SHR-mt^SHR^ (*P<0.05), *mt-CO2* was reduced ~3.6 fold in HT BN/SHR-mt^SHR^ (P<0.05), and *mt-CO3* was reduced 4.1 fold in HT BN/SHR-mt^SHR^ (P<0.05). **D:** *mt-ATP6* was reduced ~2.3 fold in HT BN/SHR—mt^SHR^ (*P<0.05), while *mt-ATP8* was reduced ~3.1 fold in HT BN/SHR-mt^SHR^ (*P<0.05). (NT: open bars; HT: closed bars).

The renal expression of five of the seven mt-encoded genes of complex I were significantly reduced in hypertensive versus normotensive BN/SHR-mt^SHR^. *mt-ND1* was reduced ~3.7 fold in HT BN/SHR-mt^SHR^ (P = 0.0317). mt-*ND3* was reduced ~2.6 fold in HT SHR/BN-mt^SHR^ (P = 0.0077). *mt-ND4* was reduced ~10.8 fold in HT SHR/BN-mt^SHR^ (P = 0.0221). *mt-ND4L* was reduced ~7.7 fold in HT SHR/BN-mt^SHR^ (P = 0.05). *mt-ND5* was reduced ~2.7 fold in HT SHR/BN-mt^SHR^ (P = 0.00167). *mt-ND6* was reduced ~1.7 fold in HT SHR/BN-mt^SHR^ (P = 0.0822) (Fig 2A). *mt-ND2* was not different between the two phenotypes (P = 0.5994).

#### Complex 3: Cytochrome bc1 complex

The renal mtRNA expression of mt-encoded cytochrome b (*mt-CYB*) was significantly reduced in hypertensive versus NT BN/SHR-mt^SHR^. *mt-CYB* was reduced ~5 fold in HT BN/SHR-mt^SHR^ (P = 0.0103) (Fig 2B).

#### Complex 4: Cytochrome C oxidase

All three mt-encoded genes of complex IV were significantly reduced in hypertensive versus normotensive BN/SHR-mt^SHR^. *mt-CO1* was reduced ~3.2 fold in HT BN/SHR-mt^SHR^ (P = 0.0164), *mt-CO2* was reduced ~3.6 fold in HT BN/SHR-mt^SHR^ (P = 0.0063), and *mt-CO3* was reduced 4.1 fold in HT BN/SHR-mt^SHR^ (P = 0.0085) (Fig 2C).

#### Complex V: ATP synthase

Two of the sixteen genes that encode vital proteins for ATP synthase of the electron transport chain are mt-encoded. *mt-ATP6* was reduced ~2.3 fold in HT BN/SHR—mt^SHR^ (P<0.05), while *mt-ATP8* was reduced ~3.1 fold in HT BN/SHR-mt^SHR^ (P = 0.0255) (Fig 2D).

#### Tissue expression in Liver and Heart

**Fig 3:**
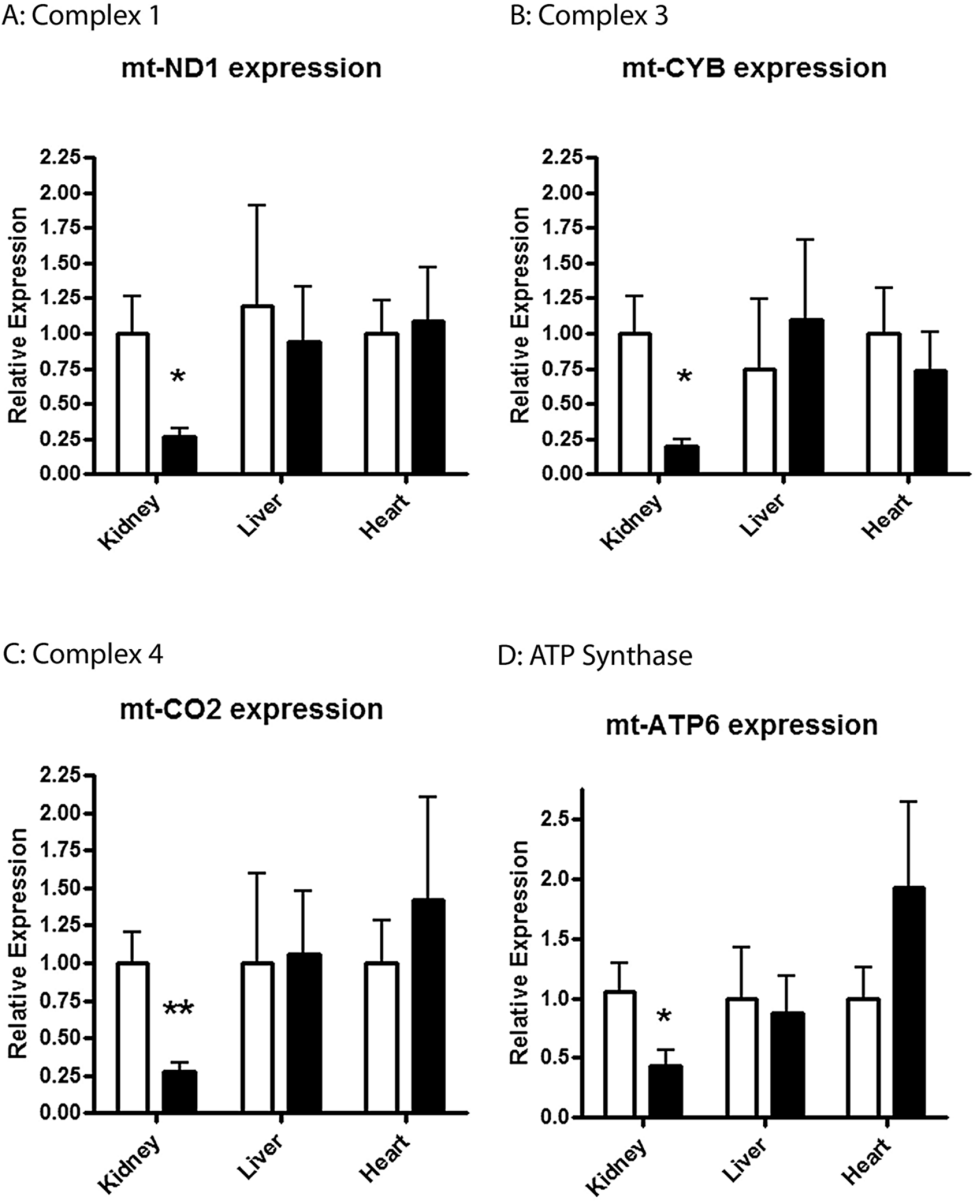
Quantitative Real-Time PCR of representative mt-genes of complex I-V were evaluated in liver and heart tissue of HT and NT BN/SHR-mt^SHR^. **A:** *mt-CYB*, **B:** *mt-CO2*, **C:** *mt-ND1*, and **D:** *ATP6* were shown to be not different (P>0.05) between the HT and NT BN/SHR-mt^SHR^ liver and heart compared with kidney tissue. (NT: open bars; HT: closed bars).

Expression levels of several mt-genes were evaluated in both the liver and heart of HT and NT BN/SHR-mt^SHR^ as described above. *mt-CYB*, *mt-CO2* and *mt-ND1*, *mt-ATP6* were shown to be not different (P>0.05) between the HT and NT BN/SHR-mt^SHR^ liver or heart tissues. This is in contrast to renal tissue wherein each of these genes exhibited reduced expression in HT vs. NT animals (Fig 3). Further, complex 1 mRNA expression was evaluated for each subunit in heart tissue, and there was no difference in mRNA expression between NT and HT animals (p>0.0.5) (S1 Fig).

### Evaluating Mitochondrial Function: Cytochrome Oxidase Activity

**Fig 4:**
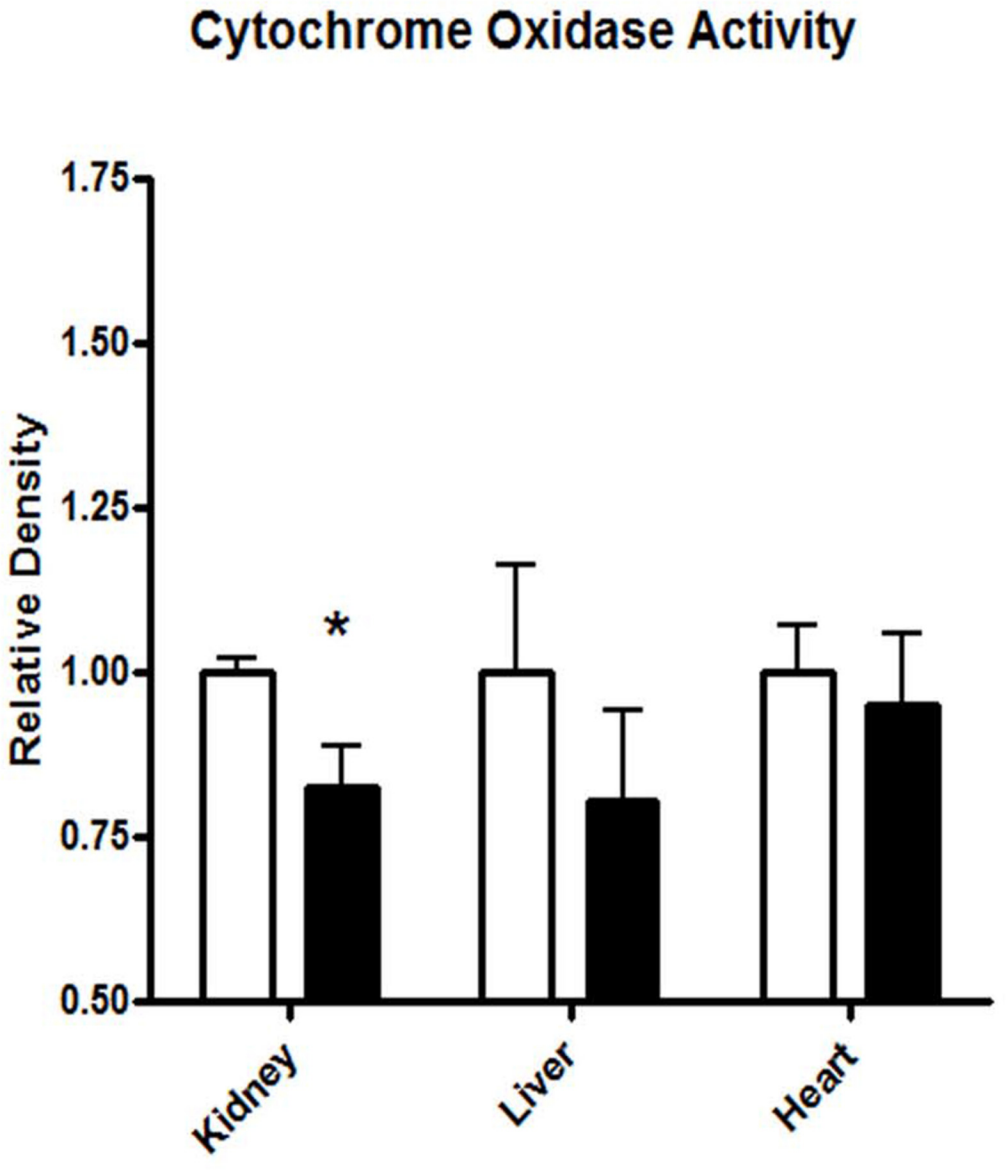
Cytochrome oxidase staining was significantly reduced in the kidney of HT vs. NT BN/SHR-mt^SHR^ (P<0.05). However, CO activity was not different in liver (p = 0.3828) or heart (p = 0.6664) of HT vs. NT BN/SHR-mt^SHR^. (NT: open bars; HT: closed bars).

CO activity was measured densitometrically in kidney, liver and heart sections (20μm) in NT (n = 6) and HT (n = 6) BN/SHR-mt^SHR^. CO activity was significantly reduced (P = 0.0269) in the kidney, but not the liver or heart (P>0.05) in HT BN/SHR-mt^SHR^ (Fig 4).

### Evaluation of Nuclear Genome Trans-Regulatory Factors

#### Peroxisome proliferator-activated receptor gamma co-activator 1-alpha(PGC-1α)

**Fig 5:**
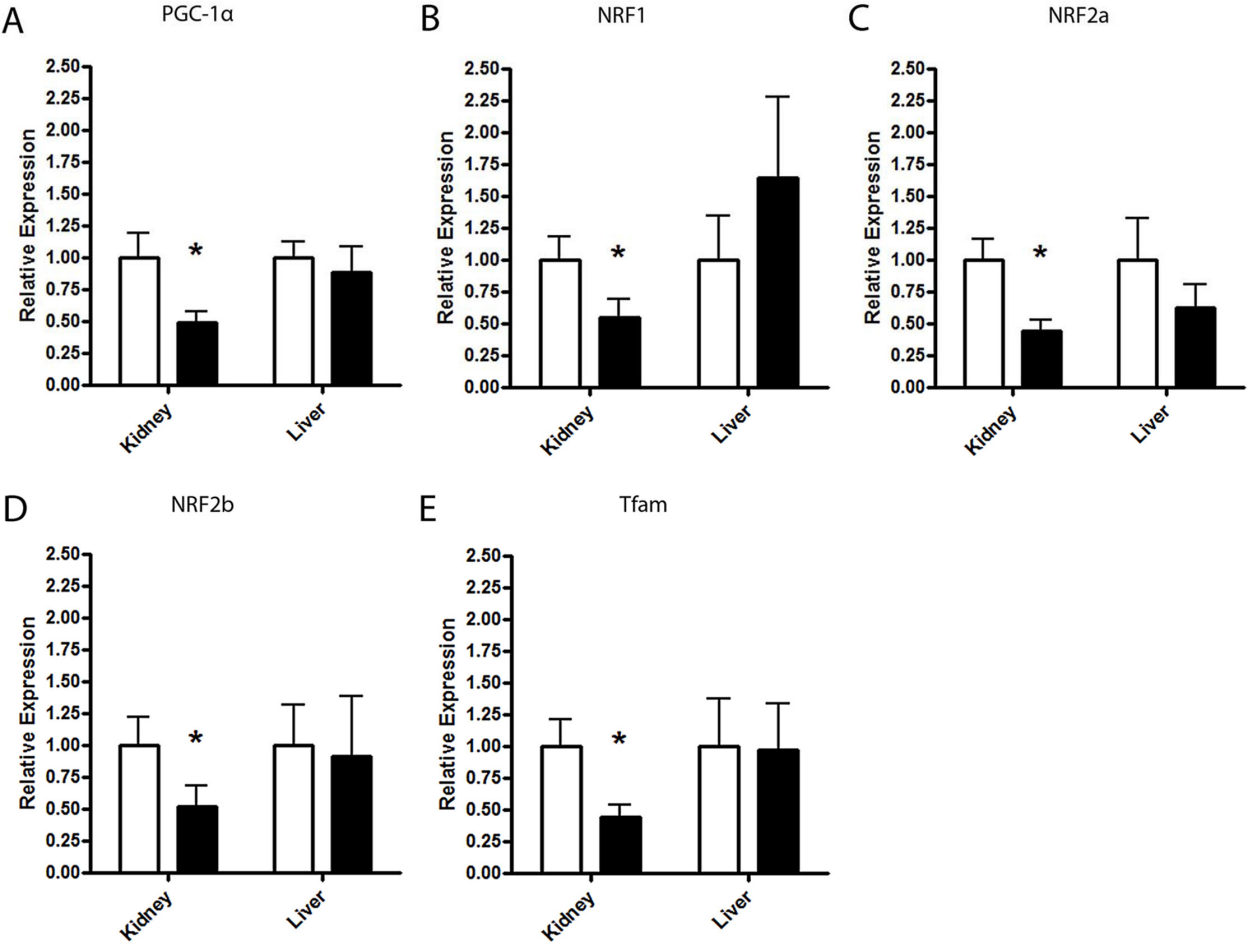
Quantitative Real-Time PCR graphs of the well-established nuclear-mitochondrial induction of mitochondrial gene transcription. **A.** *Peroxisome proliferator-activated receptor gamma coactivator 1-alpha* (*PGC-1α*). Renal *PGC-1α* mRNA expression was reduced ~2.5 fold in HT BN/SHR-mt^SHR^ compared with NT BN/SHR-mt^SHR^ (P<0.05). **B.** *Nuclear Respiration Factor (NRF) 1*. Renal *NRF1* mRNA expression was reduced ~1.8 fold in HT BN/SHR-mt^SHR^ compared with NT BN/SHR-mt^SHR^ (P<0.05). **C.** *Nuclear Respiration Factor 2A*. Renal *NRF2A* mRNA expression was reduced ~2.3 fold in HT BN/SHR-mt^SHR^ compared with NT BN/SHR-mt^SHR^ (P<0.05). **D.** *Nuclear Respiration Factor 2B*. Renal *NR2B* mRNA expression was reduced ~1.9 fold in HT BN/SHR-mt^SHR^ compared with NT BN/SHR-mt^SHR^ (P<0.05). **E.** *Mitochondrial Transcription Factor A (Tfam)*. Renal *Tfam* mRNA expression was reduced ~2.5 fold in HT BN/SHR-mt^SHR^ compared with NT BN/SHR-mt^SHR^ (P<0.05) Expression levels were not different in the liver (P>0.05) in HT BN/SHR-mt^SHR^ compared with NT BN/SHR-mt^SHR^ for any of the transcription factors. (NT: open bars; HT: closed bars).

*PGC-1α* regulates NRF-dependent transcription, increases expression of both mitochondrial and nuclear encoded genes of oxidative phosphorylation and induces mitochondrial biogenesis. HT BN/SHR-mt^SHR^ exhibited ~2.5 fold reduction in *PGC-1α* mRNA in kidney tissue compared with NT BN/SHR-mt^SHR^ (P = 0.0098) (Fig 5A).

#### Nuclear Respiratory Factors

Nuclear respiratory factors 1 and 2 are well characterized transcriptional activators of genes involved in assembly of the respiratory apparatus, as well as constituents of the mtDNA transcription and replication machinery [26]. A main factor involved in mtDNA transcription is *Tfam*, whose expression is regulated by NRF1. All three NRFs were reduced in the kidney, but not liver of HT compared to NT BN/SHR-mt^SHR^. Renal *NRF1* mRNA expression was reduced ~1.8 fold in HT BN/SHR-mt^SHR^ compared with NT BN/SHR-mt^SHR^ (P = 0.0307) (Fig 5B). Renal *NRF2a* mRNA expression was reduced ~2.3 fold in HT BN/SHR-mt^SHR^ compared with NT BN/SHR-mt^SHR^ (P = 0.0083) (Fig 5C). Renal *NRF2b* mRNA expression was reduced ~1.9 fold in HT BN/SHR-mt^SHR^ compared with NT BN/SHR-mt^SHR^ (P = 0.0377) (Fig 5D).

#### Mitochondrial Transcription Factor A (Tfam)

*Tfam* is a key activator of mammalian mitochondrial transcription. Kidney, but not liver exhibited reduced *Tfam* mRNA expression ~2.5 fold in HT BN/SHR-mt^SHR^ compared with NT BN/SHR-mt^SHR^ (P = 0.0281) (Fig 5E).

#### Nuclear-encoded mitochondrial genes: Cytochrome C-1 (Cyc1), cytochrome c oxidase, subunit Vic (Cox6c)

**Fig 6:**
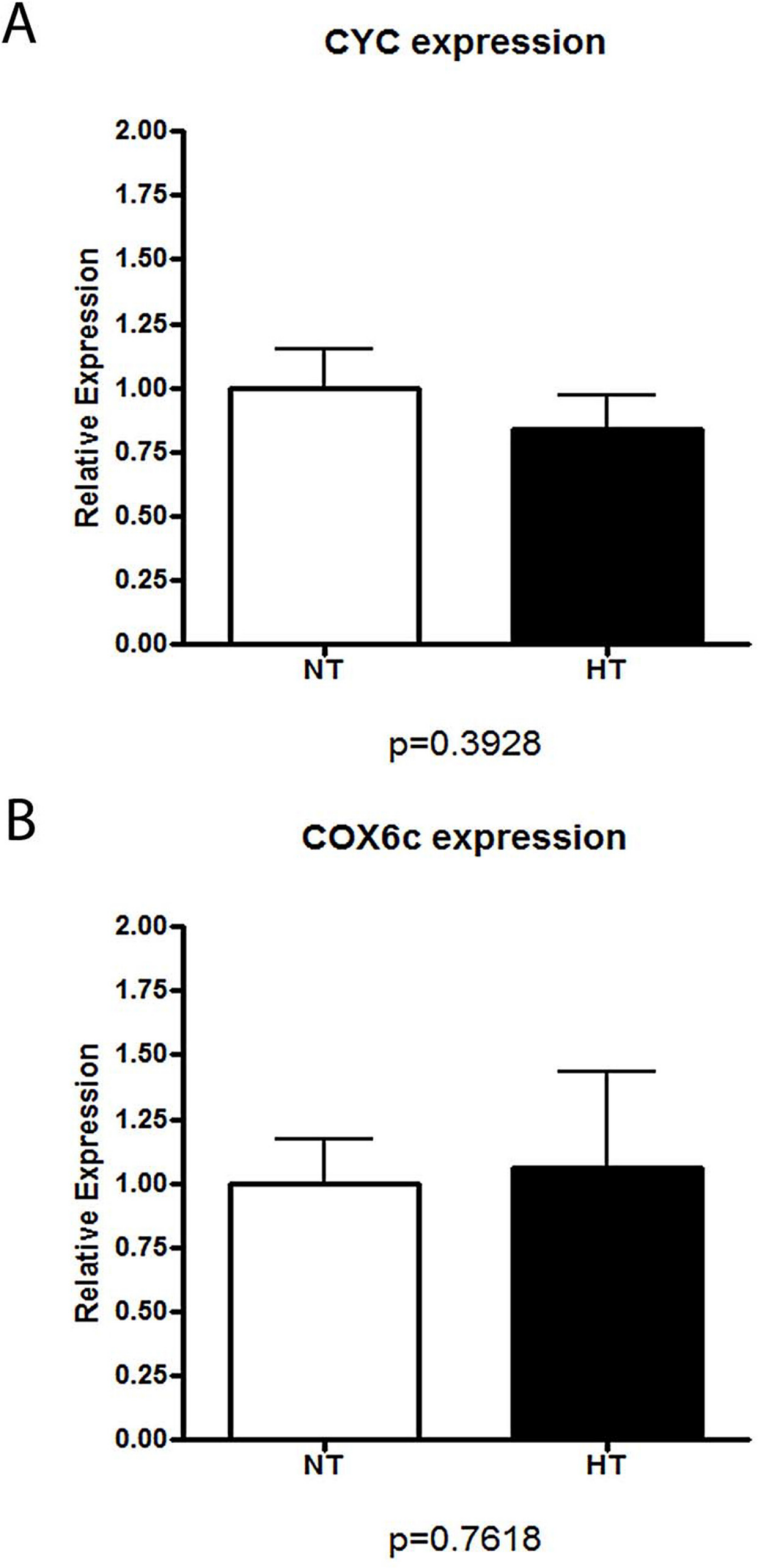
Quantitative Real-Time PCR of nuclear-encoded mitochondrial genes. Neither renal **A:** Cytochrome C-1 *(Cyc1)* (P = 0.3928) nor **B:** cytochrome c oxidase, subunit Vic *(Cox6c)*(P = 0.7618) were different between NT and HT BN/SHR-mt^SHR^. (NT: open bars; HT: closed bars).

In order to assess the downstream pathways of the nuclear encoded regulatory elements, nuclear-encoded mitochondrial gene expression were assessed. There was no difference in the renal mRNA expression of *CYC* or *Cox6c* between NT (n = 10) and HT (n = 10) BN/SHR-mt^SHR^ (P>0.05) (Fig 6).

#### Evaluating Mitochondrial Number: Citrate Synthase Assay

**Fig 7:**
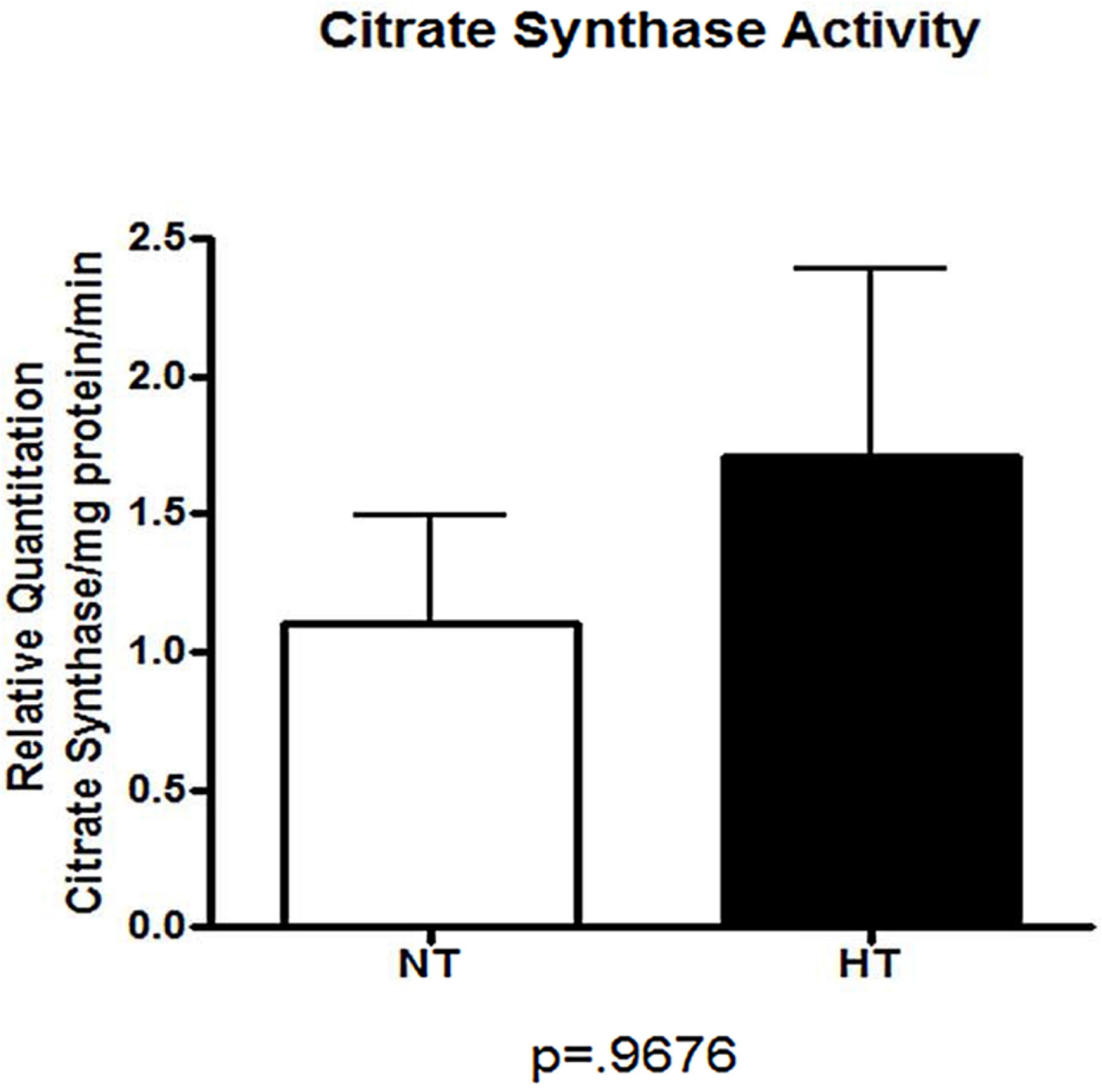
Citrate synthase activity was not different in renal tissue of NT versus HT BN/SHR-mt^SHR^. Mitochondrial number was not different between the two phenotypes (P = 0.9676). (NT: open bars; HT: closed bars).

To quantify mitochondrial number, citrate synthase activity was measured in kidney homogenates of NT (n = 10) and HT (n = 10) BN/SHR-mt^SHR^ (Fig 7). There was no difference between the two phenotypes (P = 0.9676), indicating that mitochondrial number was not driving the reduced transcript expression in HT BN/SHR-mt^SHR^ kidneys.

## Discussion

There is significant evidence the mitochondria, variations in the mitochondrial genome and in mitochondrial function may be associated with the progression of arterial hypertension [5, 7, 8, 11, 27–29]. Of particular interest is the nature of mitochondrial inheritance, being strictly maternal, and of female origin. The results of the current study provide evidence that gene expression in renal cortical mitochondrial genes encoding respiratory chain complexes is downregulated in hypertensive rats stemming from a SHR/BN conplastic-breeding paradigm. This breeding method provided a continuous passing of the maternal SHR mitochondrial genome, while mixing the inherited nuclear genome between the maternal SHR and paternal BN with each succeeding generation. We have reported the maintenance of arterial systolic hypertension for 6 consecutive generations despite the reduction in the maternally derived nuclear genome (Fig 1) [18]. Results from the current study show mitochondrial protein-coding genes critical for OXPHOS exhibited significantly reduced expression in kidney cortex, but not in the liver or heart, in HT BN/SHR-mt^SHR^ compared with NT BN/SHR-mt^SHR^ (Figs 2 and 3). In HT BN/SHR-mt^SHR^, nuclear genes involved in mitochondrial biogenesis and transcription (*PGC-1α*, *NRF1*, *NRF2a/b*, *Tfam*), also exhibited reduced expression in kidney cortex, but not liver, compared with NT BN/SHR-mt^SHR^ (Fig 5). Thus, in hypertensive rats with SHR mitochondrial genome but minimal SHR nuclear genome, there is kidney-specific reduction in expression of both mitochondrial and nuclear genes critical to OXPHOS.

The viability of individual electron transport chain (ETC) subunits and the potential for disease has been previously evaluated [28, 30]. Lopez-Campistrous *et al*. [28] reported that defects in complex I in the brainstem of SHR increased reactive oxygen species production, decreased ATP synthesis and impaired respiration in hypertension. We report here that reduced expression of the mt-genes coding for complex I, which may potentially reduce complex I function. Altered function of complex III, a major site of superoxide formation and reactive oxygen species (ROS) production, may play an important role in renal mitochondrial ETC dysfunction. Das *et al*. [31] reported that the regulation of ATP synthase is abnormal in SHR cardiac cells, as demonstrated by the inability to respond to acute increases in energy demand compared to cells from normotensive rats. Data from our study indicate that mt-gene expression is reduced in kidneys, a key organ in blood pressure control. To evaluate mitochondrial function, we measured cytochrome oxidase histochemical staining in NT and HT BN/SHR-mt^SHR^. Cytochrome oxidase serves as an endogenous metabolic marker. As shown in Fig 4, CO staining was significantly reduced in the kidney cortex, but not in liver or heart of HT BN/SHR-mt^SHR^. Thus, oxidative phosphorylation may be reduced in the kidneys of HT BN/SHR-mt^SHR^ which is consistent with the renal cortical-specific downregulation of mitochondrial gene expression. Taken together, the altered function of ETC subunits derived from decreased kidney cortex-specific mt-gene expression may have several implications to the progression of hypertension.

Data presented here poses the important and critical question: Does the reduction of renal mitochondrial gene expression and function contribute significantly to the etiology of hypertension? Or does hypertension cause the downregulation of mitochondrial gene transcription and function? Recently, Lee *et al*. [32] postulated that increased mitochondrial activity in proximal convoluted tubule cells of young, normotensive SHR may contribute to the development of hypertension at adulthood. These studies show that various parameters of mitochondrial activity were elevated in very young SHR prior to the onset of hypertension, while mt-gene expression remained unchanged. Our study documents mitochondrial genes encoding proteins of each mitochondrial subunit of mRNA were reduced in renal tissue of HT BN/SHR-mt^SHR^ compared with NT BN/SHR-mt^SHR^. It is possible that mitochondrial activity is elevated in renal proximal tubules of very young SHR prior to the onset of hypertension. As the development of hypertension progresses in the maturing SHR, renal mitochondrial gene expression may then decline as a compensatory mechanism to, for instance, decrease sodium transport along various segments of the nephron. This is plausible, as altered renal sodium handling and renal function are a hallmark of hypertension. Ongoing and future studies are being conducted to address this possible relationship and mechanism specific to the SHR.

One of the more interesting aspects of this study is a potential ETC dysfunction driven by transcript differences in the kidneys, but not in other tissues, of mt-genes and the nuclear *trans*-factors that regulate them. As shown in Fig 4, CO histochemical staining was significantly reduced in the kidneys, but not liver or heart of HT BN/SHR-mt^SHR^, hence, it appears that mitochondrial function was decreased in the kidneys of HT BN/SHR-mt^SHR^. Altered renal function has been well recognized as a key factor in the development and maintenance of hypertension [13, 16, 33–38]. One such mechanism that may be responsible is altered regulation of the renin-angiotensin system (RAS). The interactions among RAS and altered mitochondrial function has been advanced recently by Benigni *et al*. [39] Deletion of the *Agtr1a* gene resulted in the reduced age-related cardio-renal complications, improved mitochondrial biogenesis, and increased longevity in mice. Treatment with antioxidants, mitochondrial superoxide dismutase mimetics, and AT_1_r blockers decreased vascular O_2_^**.-**^ production and attenuated development of hypertension in SHR [40, 41]. In this regard, we have previously shown that renal cortex of HT BN/SHR-mt^SHR^ exhibit elevated AT_1_r mRNA (*Agtr1a*) expression compared to NT BN/SHR-mt^SHR^, while the renal and systemic expression of renin, angiotensinogen, and angiotensin-converting enzymes were not different [18]. Renal cortex of HT BN/SHR-mt^SHR^ also had elevated AT_1_r protein compared with NT BN/SHR-mt^SHR^, and this increase was positively correlated with elevated systolic BP [18]. De Cavanagh *et al*. [27] demonstrated that oxidative stress is associated with mitochondrial dysfunction in SHR, and that this dysfunction is attenuated with AT_1_r blockade with Losartan. These data support the hypothesis that hypertensive phenotype derived from the SHR is driving the reduced mitochondrial gene expression, which may lead to decreased OXPHOS.

**Fig 8:**
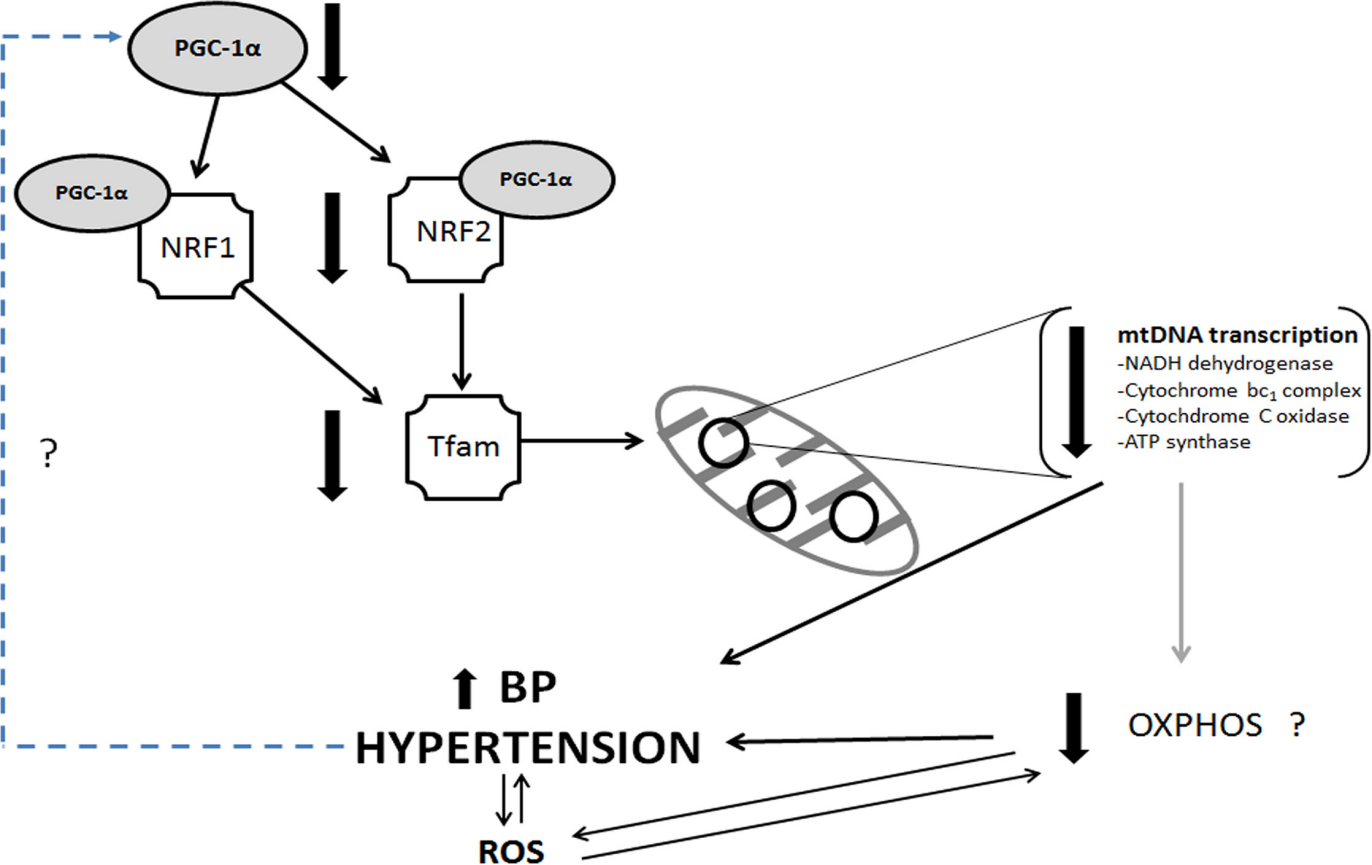
Hypothetical nuclear-mitochondrial pathway driving reduced mtDNA transcription, ultimately leading to elevated arterial pressure and hypertension. The pathway is clearly reduced in the kidney, but not liver of HT BN/SHR-mt^SHR^ compared with NT BN/SHR-mt^SHR^.

*PGC-1α* plays a central role in regulating mitochondrial content and function within cells, because of its ability to co-activate and augment several promoters of nuclear-encoded mitochondrial genes, as well as regulating mitochondrial transcription via the *NRF*-*Tfam* pathway [42]. *PGC-1α* regulates NRF-dependent transcription, increases expression of both mitochondrial and nuclear encoded genes of oxidative phosphorylation and induces mitochondrial biogenesis [43]. It has been shown that metabolic functions are controlled by *PGC-1α* in a tissue specific manner in brown fat, muscle and liver [44], however kidney regulation of *PGC-1α* and its effectors is unknown. Results from this study show a coordinated reduction of the kidney-specific expression of nuclear and mitochondrial genes vital to OXPHOS in hypertensive BN/SHR-mt^SHR^, which may play a significant role in the development and maintenance of hypertension (Fig 8).

In order to further assess the downstream pathways of the nuclear-encoded regulatory elements, the expressions of two separate nuclear-encoded mitochondrial genes were assessed. Other nuclear-encoded mitochondrial genes known to be regulated by the *PGC-1α-NRF-Tfam* pathway were not different. Neither kidney Cytochrome C-1 *(Cyc1)* nor cytochrome c oxidase subunit Vic *(Cox6c)* were differentially expressed in kidney cortex of HT and NT BN/SHR-mt^SHR^ (Fig 6). This suggests that the biogenesis and transcription of mitochondrial genes, known to be regulated by the *PGC-1α* pathway, may be regulated in a more complex manner than previously thought. Mitochondrial gene expression also appears to be regulated in a tissue-specific manner. If this is the case, our data suggests that tissues of high metabolic activity such as the kidney, may have a critical role in the development of—or response to—hypertension.

Whenever mitochondrial disturbances inhibit electron transport, electrons are forwarded into an increased generation of ROS [9]. Increased mitochondrial ROS is linked to metabolic diseases such as aging, diabetes and hypertension [45–47]. Mitochondria are a major site of oxygen consumption and oxidative stress due to generation of ROS, where complexes I and III are the main sites of mitochondrial superoxide formation [48]. Recently, mitochondrial ETC dysfunction has been shown to directly cause oxidative stress during hypertension [6]. Ballinger *et al*. [49] have demonstrated that reactive oxygen species decreased mtRNA transcripts, mitochondrial protein synthesis and reduced cellular ATP levels. Taken together, ROS decreases OXPHOS, and decreased OXPHOS increases ROS, both of which contribute to hypertension.

## Conclusion

In summary, we present novel data documenting a decrease in a well-defined nDNA-mtDNA interactive pathway resulting in decreased mtDNA transcripts of proteins vital to OXPHOS in renal cortex. This potential coordinated reduction of nuclear-mitochondrial OXPHOS genes and its association with hypertension until now was largely unexplored (Fig 8). Though the exact mechanisms driving this reduction in gene expression is currently not known, it is clear that reduced mt-gene expression in the kidney coincides with hypertension in adult BN/SHR-mt^SHR^. Using our current model, where the nuclear genome is increasingly BN while maintaining the SHR mitochondrial genome, in combination with other similar models, the relevance of the specific control of each of these genomes and how they may contribute to disease may be revealed. Furthermore, the nuclear-mitochondrial gene expression interactions may be critically important in the manifestation of the renal disease process and progression of hypertension.

## Supporting Information

S1 Fig Quantitative Real-Time PCR of representative mt-genes of complex I were evaluated in heart tissue of HT and NT BN/SHR-mt^SHR^. None of the 7 mitochondrial encoded genes of complex 1 exhibited gene expression differences (P>0.05) between the HT and NT BN/SHR-mt^SHR^ (NT: open bars; HT: closed bars).
(TIF)

S2 Fig Quantitative Real-Time PCR of superoxide dismutases. Neither renal **A:** Superoxide dismutase 1 (SOD1) nor **B:** superoxide dismutase 2 (SOD2) were different (P>0.05) between NT and HT BN/SHR-mt^SHR^ (NT: open bars; HT: closed bars).
(TIF)
