# Supplementary figures and images for: Kidney-Specific Reduction of Oxidative Phosphorylation Genes Derived from Spontaneously Hypertensive Rat
Source: PLoS One. 2015 Aug 26;10(8):e0136441. doi: 10.1371/journal.pone.0136441 (PMC4550288; doi:10.1371/journal.pone.0136441)

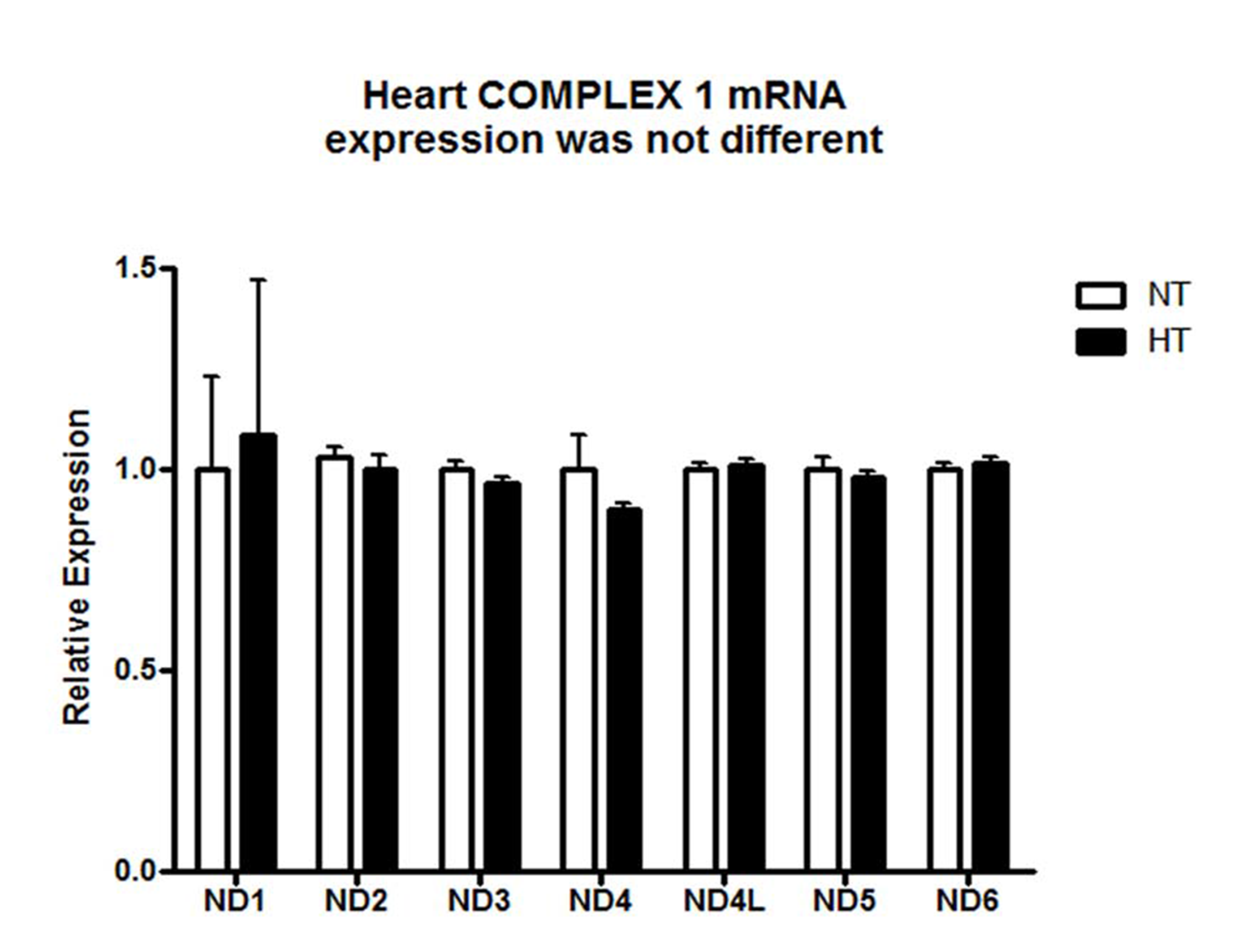

Supplement: S1 Fig — None of the 7 mitochondrial encoded genes of complex 1 exhibited gene expression differences (P>0.05) between the HT and NT BN/SHR-mtSHR (NT: open bars; HT: closed bars). (TIF) [file pone.0136441.s001.tif]

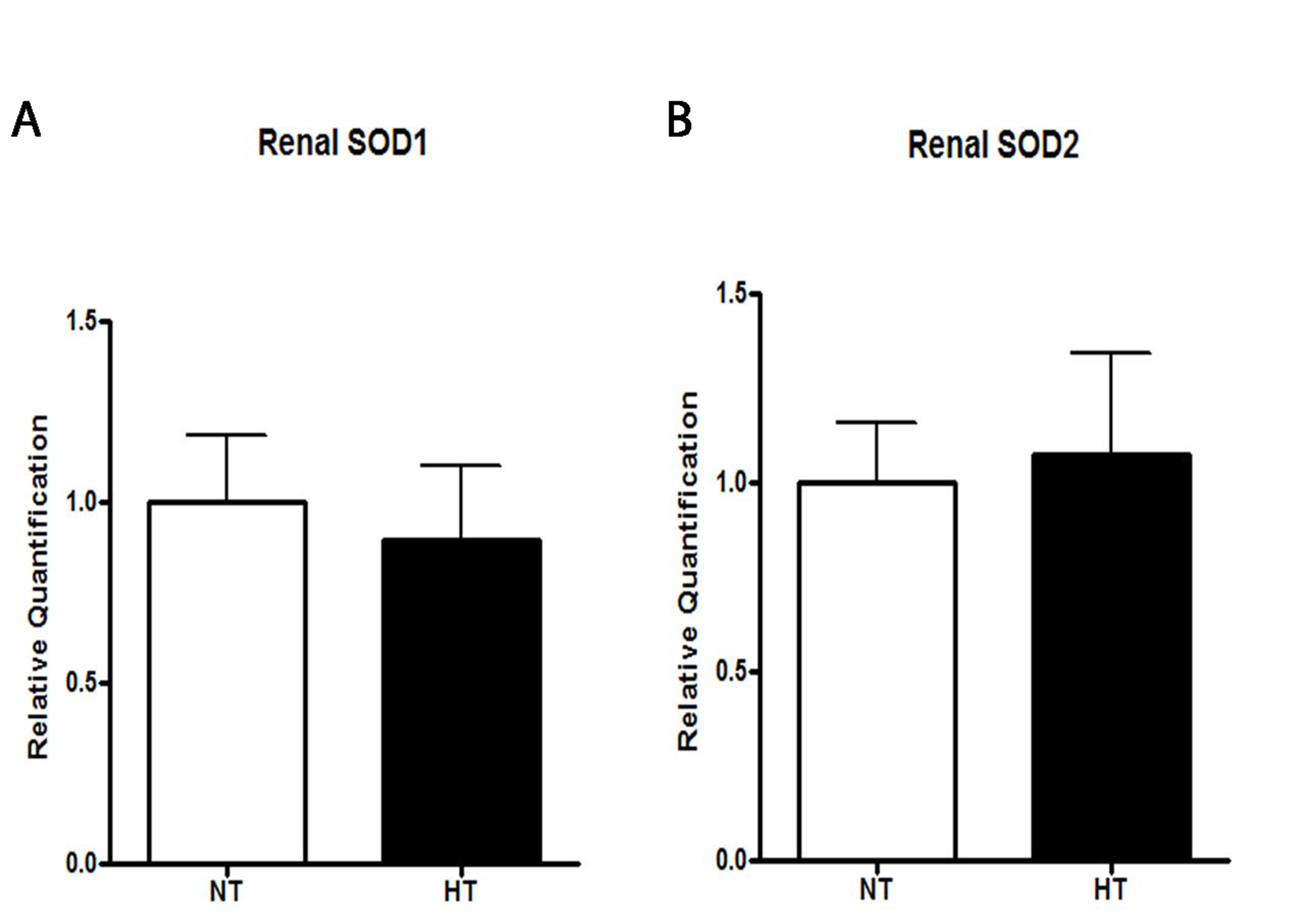

Supplement: S2 Fig — Neither renal A: Superoxide dismutase 1 (SOD1) nor B: superoxide dismutase 2 (SOD2) were different (P>0.05) between NT and HT BN/SHR-mtSHR (NT: open bars; HT: closed bars). (TIF) [file pone.0136441.s002.tif]
